# Efficacy of combined oral hygiene devices for interproximal plaque removal: an in vitro study

**DOI:** 10.1186/s12903-026-08794-6

**Published:** 2026-06-08

**Authors:** Ryoko Otsuka, Ayako Okada, Nao Suzuki, Masahiro Nakano, Hiromi Uematsu, Toyotaro Sugawara, Emi Ishiguro, Nobuhiro Hanada, Yasuko Momoi, Chikahiro Ohkubo

**Affiliations:** 1 Department of Oral Rehabilitation and Prosthodontics, Tsurumi University School of Dental Medicine, Yokohama, Japan; 2 Department of Preventive and Public Health Dentistry, Fukuoka Dental College, 2-15-1, Tamura, Sawara-ku, Fukuoka, 814-0193 Japan; 3 Oral Medicine Research Center, Fukuoka Dental College, Fukuoka, Japan; 4 Nakano Dental Clinic, Ota, Japan; 5 Division of Dental Hygiene, The Nippon Dental University Hospital, Chiyoda, Japan; 6 Sugawara Dental Office, Soka, Japan; 7 Ishiguro Dental Clinic, Ichikawa, Japan; 8 Tsurumi University, Yokohama, Japan; 9 Institute of Photochemistry and Photofunctional Materials, University of Shanghai for Science and Technology, Shanghai, China

**Keywords:** Plaque removal, Interdental brush, Single-tufted brush, Dental floss

## Abstract

**Background:**

This study compared interproximal plaque removal rates when oral hygiene devices were used with a manual toothbrush.

**Methods:**

Artificial plaque was applied to the mesial interproximal surface of a lower left first molar mounted on a jaw model within a simulator. Removal was compared among 10 groups, including toothbrush alone (TB) and combinations of single-tufted brushes (Tuft), interdental brushes (IDB), and dental floss (string floss [SF] or holder-type floss [HF]). Five dental professionals performed the procedures. Pre- and post-cleaning images were analyzed to determine removal rates. Statistical analysis was conducted using a linear mixed-effects model with Tukey’s post-hoc test.

**Results:**

The estimated marginal mean (EMM) for the TB group was 0.44 (95% confidence interval [CI]: 0.37–0.50). All combinations achieved significantly higher removal rates (*p* < 0.001). The TB, IDB, and SF combination achieved the highest EMM (0.93; 95% CI: 0.87–0.99). No significant differences were observed among the three SF combinations; however, all three demonstrated significantly higher rates than other protocols (*p* < 0.001).

**Conclusions:**

Adjunctive devices significantly improve interproximal plaque control compared to toothbrushing alone. Under the conditions of this experimental model, the combinations that included string floss demonstrated the highest efficacy for physical plaque removal. Further studies are needed in different anatomical settings and broader user populations.

## Background

Oral diseases, including dental caries and periodontal disease, remain significant global health challenges that affect nearly half of the world’s population. According to the Global Burden of Disease (GBD) 2021 Oral Disorders Collaborators, severe periodontal disease and untreated caries affected approximately 3.69 billion people as of 2021. Notably, prevalence and disability-adjusted life years have remained largely unchanged over the past 30 years [1], implying that conventional oral hygiene measures have not yet achieved sufficient success. This issue is further compounded by the bidirectional relationship between oral and non-communicable diseases, in which promoting oral health contributes significantly to systemic well-being [2]. Effective plaque removal is therefore essential, as thorough control prevents tooth loss and avoids associated risks such as masticatory dysfunction, deteriorated nutritional status, and a decline in quality of life [3, 4].

While daily self-care with a toothbrush remains the fundamental method of plaque control [5], achieving efficient interproximal plaque removal with a manual toothbrush alone remains a challenge. Although manufacturers have attempted to improve efficacy by refining toothbrush designs, these efforts have yielded limited results. In a previous study, we reported that the average interproximal plaque removal rate for 26 commercially available manual toothbrushes was less than 50%. Furthermore, we found that these rates remained insufficient regardless of variation in toothbrush morphology, such as bristle stiffness, tuft count, or total head length [6].

To address the limitations of toothbrushing alone, the use of interdental cleaning devices, such as dental floss and interdental brushes (IDBs), is widely recommended.

Effective oral health strategies require evidence-based guidance regarding the device combinations that provide optimal cleaning efficacy. Although systematic reviews have demonstrated that IDBs are more effective than dental floss for reducing plaque and gingivitis, several studies have indicated that floss may be more suitable for tight interdental spaces [7–9]. In addition, previous studies have suggested that differences in oral hygiene protocols, including the sequence of procedures, may influence plaque removal efficacy and fluoride retention; however, findings remain inconsistent [10–12]. Conversely, patient-level studies of interdental cleaning have emphasized a discrepancy between the theoretical efficacy of these devices and their actual clinical effectiveness in routine self-care. A Cochrane review by Worthington et al. [13] reported that the use of floss or IDBs in addition to toothbrushing may reduce gingivitis and plaque compared to toothbrushing alone; however, the certainty of the evidence is low, and the clinical relevance of the observed effects remains limited. Patient motivation, adherence, technique, and ease of use significantly influence the implementation and clinical effectiveness of interdental cleaning devices [14], and the prevalence of interdental cleaning remains suboptimal. Although the 2024 Survey on the Status of Dental Diseases in Japan reported an interdental cleaning rate of 54.2%, significant disparities persist across age and sex strata, and the establishment of a consistent habit remains challenging [15]. Furthermore, clinical studies are influenced by multiple confounding factors—such as patient background, proficiency, compliance, salivary conditions, and gingival inflammation—which limit objective comparison of the intrinsic physical access and cleaning performance of the devices. Previous in vitro studies, conducted under standardized and reproducible conditions, have provided detailed evaluations of the mechanical performance of various interdental cleaning devices [16–19]. These studies offer important foundational data regarding the efficacy of individual devices. Although toothbrushing and interdental cleaning instruments are commonly used in combination during daily oral hygiene practice, systematic studies regarding the effectiveness of combined cleaning protocols remain limited. We systematically evaluated 10 combined protocols using a manual toothbrush together with interdental cleaning instruments under standardized in vitro conditions. Furthermore, a linear mixed-effects model was applied to control for inter-operator variability, enabling estimation of the effects attributable to the cleaning protocols.

## Materials and methods

### Oral care tools

The cleaning aids used in our study are summarized in Table 1. Products were selected based on findings from previous studies using similar artificial plaque models [6].

**Table 1:** Comparison of key characteristics of oral hygiene devices

Device Class	Device	Stiffness	Type	Length (mm)	Diameter (mm)	Size	Filament thickness (µm)	Filament number	Wax attachment
Brush	Toothbrush	Normal	Flat	9.0	0.17	NR	NA	NA	NA
	Single-tufted brush	Soft	Cone	4.5–8.5	0.085	NR	NA	NA	NA
	Interdental brush	NR	Cylinder	8.0	NR	ISO 2 (wire size: 0.5 mm, bristle diameter: 2.5 mm)	NA	NA	NA
Floss	String floss	NA	NA	NA	NA	NA	NR	1,400	Unwaxed
	Y-shaped holder-type dental floss	NA	NA	NA	NA	NA	3.8	50	Unwaxed

Products used in this study: Toothbrush: OralCare, tuft24 MS, Single-tufted brush: Sunstar, BUTLER SINGLE TUFT #01S, Interdental brush: TePe Oral Health Care, Original inter dental brush, String floss: GC Corporation, rucello floss (Unwaxed) and Y-shaped holder-type dental floss: Lion Corporation, DENT.EX Ultra floss S

*NA* Not applicable, *NR* Not reported

### Experimental plaque model

**Fig. 1:**
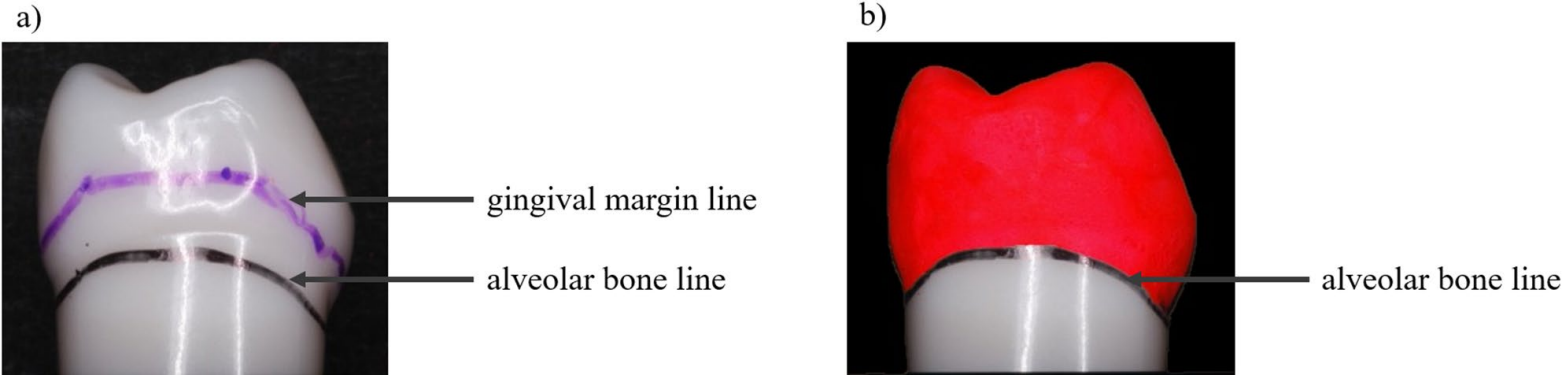
Proximal surface of the first molar prosthesis before testing. **a** Before coating with artificial plaque (test surface). Illustration of the gingival margin and alveolar bone crest at the proximal surface of a prosthetic tooth. **b** Coated with artificial plaque before brushing

The experimental setup is illustrated in Fig. 1. The artificial left mandibular first molar was removed from a dental simulator jaw model (D18FE-500E, Nissin Dental Products Co., Ltd., Kyoto, Japan), and the adjacent tooth surfaces were prepared for coating with a nail-polish-type artificial plaque (Nissin Dental Products Co., Ltd.; Fig. 1) [6, 20–22]. The material is a quick-drying, resin-based liquid intended for dental training applications. After drying, it adheres firmly to the tooth surface and can be mechanically removed during brushing simulation. To minimize variability in plaque thickness, all application procedures were performed by a single operator using a standardized technique across all trials. In addition, to ensure that morphological differences inherent to specific teeth did not influence the findings, melamine teeth (artificial teeth) were selected randomly by each operator from a storage box and reattached to the jaw model.

### Plaque removal test

Ten experimental protocols were established to evaluate various combinations of oral hygiene devices. These included toothbrushing alone (TB), four dual-combination groups (TB combined with a single-tufted brush [Tuft], IDB, string floss [SF], or Y-shaped holder floss [HF]), and five triple-combination groups (TB combined with Tuft or IDB in addition to one of the two floss types). Our study was conducted by five oral health professionals from Tsurumi University, including four dentists with 3–7 years of postgraduation experience and one dental hygienist with 11 years of experience. Before the experiment, calibration procedures were performed among the five operators. Operator positioning was standardized to the 12 o’clock position. The scrubbing method was selected because it provides high procedural reproducibility in in vitro experiments using phantom models, minimizes inter-operator variability, and has been adopted in similar previous studies [6]. The toothbrush bristles were applied vertically to the artificial teeth, and the buccal and lingual surfaces were brushed for 15 s each (30 strokes with a stroke length of 10 mm). In our study, toothbrushing was standardized as a baseline mechanical cleaning procedure targeting the areas adjacent to the contact points rather than the central interproximal surfaces directly. Brushing pressure was determined based on measurements obtained from a single operator and standardized across all participants. When a Tuft was used, three mesio-distal strokes were performed along the cervical margin on both sides, followed by five circular motions within the interdental space. The IDB was inserted from the buccal side and brought into contact with the mesial surface of the first molar and the distal surface of the second premolar, and was moved back and forth three times in each direction.

For the flossing procedure, approximately 40 cm of SF was wrapped around the middle finger to maintain a working length of 1–1.5 cm. The floss was passed through the contact point using a sawing motion, positioned subgingivally along the interdental surface, and swept coronally once. Y-shaped holder floss was used with the same technique.

Regarding the order of the experimental protocols, and taking into account the operators’ experience and professional roles, three operators were assigned initially to the TB group, whereas two operators were assigned to the group involving auxiliary cleaning devices. Operators assigned to the TB-start group first performed the TB protocol and then proceeded sequentially to the dual-combination protocols (toothbrush followed by an additional cleaning device) and the triple-combination protocols (toothbrush followed by two adjunctive devices). Subsequently, operators assigned to the combination groups performed the triple-combination protocols in the following sequence: toothbrush first, followed by Tuft or IDB, and finally SF or HF; next, they completed the TB protocol. The order of procedures within each group was determined independently by each operator and was not disclosed to the other operators. In the multi-tool protocols, the model was not rinsed between sequential cleaning procedures. This series of protocols was repeated five times as one cycle, resulting in 50 procedures per operator.

### Measurement of plaque removal area

Following the cleaning procedures, the artificial tooth was removed from the jaw model, secured in a mold for documentation, and mounted on a copy stand at a fixed distance of 30 cm under indoor fluorescent lighting with a ring flash (exposure time: 1/125 s; ISO: 200). Images were analyzed using Adobe Photoshop CS5 Extended (Adobe Systems Incorporated, San Jose, CA, USA). To extract red-stained regions in the pre-cleaning images, the fixed “Reds” preset of the Color Range function was applied uniformly to all images without adjustment of threshold settings. To align pre- and postcleaning images, the precleaning image was converted into an inverted reference layer and superimposed onto the corresponding postcleaning image. Minor angular deviations during image acquisition (within approximately ± 1°) were corrected to improve alignment accuracy.

Background noise and isolated pixels were corrected by a single examiner using predefined, standardized criteria. In addition, correction procedures were standardized to minimize artifacts arising from removal and repositioning of the model during image acquisition. Considering that identical analysis settings and standardized procedures were applied uniformly to all images, subjective variability in image processing was minimized.

The extent of plaque removal was quantified by counting pixels in each image, and the final removal rate was calculated as the ratio of the non-coated area after brushing to the total coated area before brushing.

To ensure the objectivity of the evaluation, all operator data were anonymized using identification numbers prior to statistical analysis. Only identification numbers were assigned to the images, and these identifiers were not disclosed to the image analyst. All image analyses were performed by a single blinded examiner using standardized analysis settings.

### Statistical analysis

Statistical analysis was performed by a single statistician who was independent of the investigators responsible for trial conduct and image analysis. Analyses was conducted using the R environment (version 4.3.1; The R Foundation for Statistical Computing, Vienna, Austria) via the EZR interface (version 1.68; Saitama Medical Center, Jichi Medical.

University, Saitama, Japan). The primary stages of statistical model construction and analysis were performed using the R lme4 package (version 1.1.38), whereas the emmeans package (version 2.0.3) was used to estimate marginal means (EMMs) and conduct multiple pairwise comparisons using Tukey’s adjustment.

Our study was designed as an exploratory in vitro study to compare multiple oral hygiene protocols under standardized experimental conditions. Therefore, no a priori sample size calculation based on prior power analysis was performed. However, in reference to a previous study using a similar artificial plaque model, five replicates were conducted for each protocol across five operators, and a repeated-measures design was adopted [6]. To account for inter-operator technical variability and the hierarchical structure of repeated observations, data were analyzed using a linear mixed-effects model. The model was specified as follows: removal.rate ~ factor(method) + (1 | ID).

The fixed effect was the cleaning method (10 protocols), and the random effect was the operator (ID; 5 dental professionals) with a random intercept. This approach enabled adjustment for inter-operator variability in technical performance. Residual diagnostics were performed to assess model fit. Normality of residuals was evaluated using Q-Q plots, and homoscedasticity was assessed visually using residual-versus-fitted plots. No extreme outliers were identified. Although the outcome variable was bounded between 0 and 1, the residual distribution and variance structure did not indicate substantial violations of the assumptions underlying linear mixed-effects modeling.

## Results

**Fig. 2:**
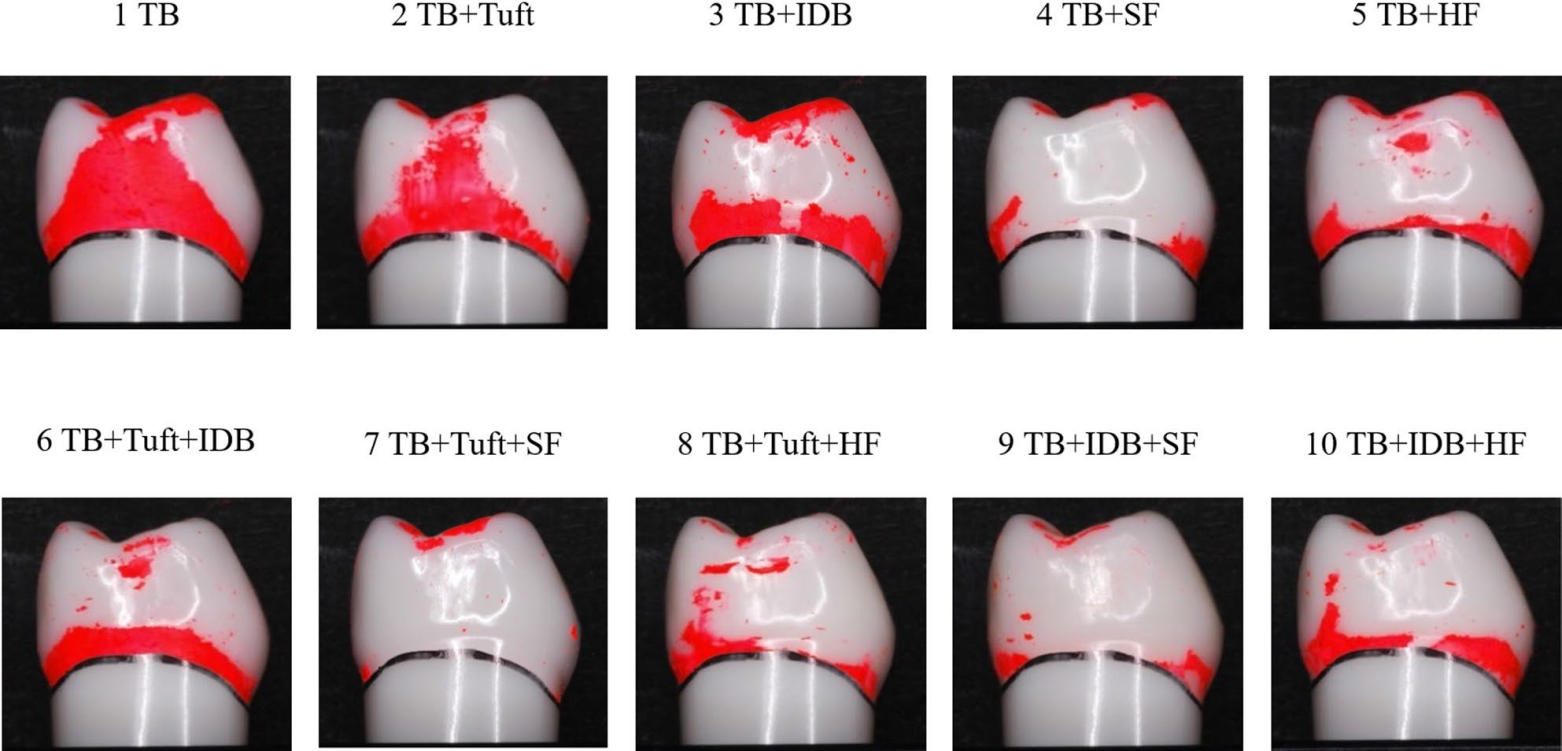
Proximal surface after testing. Ten removing protocols were established based on the presence and type of oral hygiene devices used: 1. TB (toothbrush alone). 2. TB + Tuft (single-tufted brush). 3. TB + IDB (interdental brush). 4. TB + SF (string floss). 5. TB + HF (Y‑shaped holder floss). 6. TB + Tuft + IDB. 7. TB + Tuft + SF. 8. TB + Tuft + HF. 9. TB + IDB + SF. 10. TB + IDB + HF

Representative interproximal views of the artificial plaque before and after removal are presented in Figs. 1 and 2. In the toothbrush alone (TB) and TB + single-tufted brush (Tuft) groups, significant plaque remained in areas extending from the contact point to the gingival margin, indicating insufficient cleaning. Conversely, plaque removal was clearly observed directly beneath the contact point in the combinations involving IDB or Y-shaped holder floss (HF). The most comprehensive cleaning, covering both the area near the contact point and the gingival margin, was achieved in the groups utilizing SF. Notably, some residual plaque remained near the bucco-lingual gingival margins in several HF combinations.

The quantitative results of the plaque removal tests are summarized in Table 2. The EMM for the baseline TB group was 0.44 (95% confidence interval [CI]: 0.37–0.50). All combination groups demonstrated significantly higher plaque removal rates than the TB group (Tukey-adjusted *p* < 0.001). The analysis revealed minimal variance due to operator differences (0.0025) and a residual variance of 0.003999, implying high consistency across the five operators.

**Table 2:** Means and EMMs of plaque removal rates by oral care tool

	Oral hygiene device combinations	Means ± SD	EMMs (95% CI)	*P*-value (vs.TB)
1	TB (control)	0.44 ± 0.07	0.44 (0.37–0.50)	―
2	TB + Tuft	0.61 ± 0.07	0.61 (0.55–0.67)	< 0.001
3	TB + IDB	0.69 ± 0.07	0.69 (0.63–0.75)	< 0.001
4	TB + SF	0.91 ± 0.08	0.91 (0.85–0.97)	< 0.001
5	TB + HF	0.84 ± 0.10	0.84 (0.78–0.88)	< 0.001
6	TB + Tuft + IDB	0.75 ± 0.07	0.75 (0.69–0.81)	< 0.001
7	TB +Tuft + SF	0.92 ± 0.07	0.92 (0.86–0.98)	< 0.001
8	TB + Tuft + HF	0.85 ± 0.10	0.85 (0.79–0.91)	< 0.001
9	TB + IDB + SF	0.93 ± 0.06	0.93 (0.87–0.99)	< 0.001
10	TB + IDB + HF	0.83 ± 0.09	0.83 (0.77–0.90)	< 0.001

P-values are adjusted for multiple comparisons using the Tukey method. The reference group for the comparison is TB

*Abbreviations*: *TB *toothbrush alone, *Tuft *single‑tufted brush, *IDB* interdental brush, *SF *string floss, and *HF* Y‑shaped holder floss

**Fig. 3:**
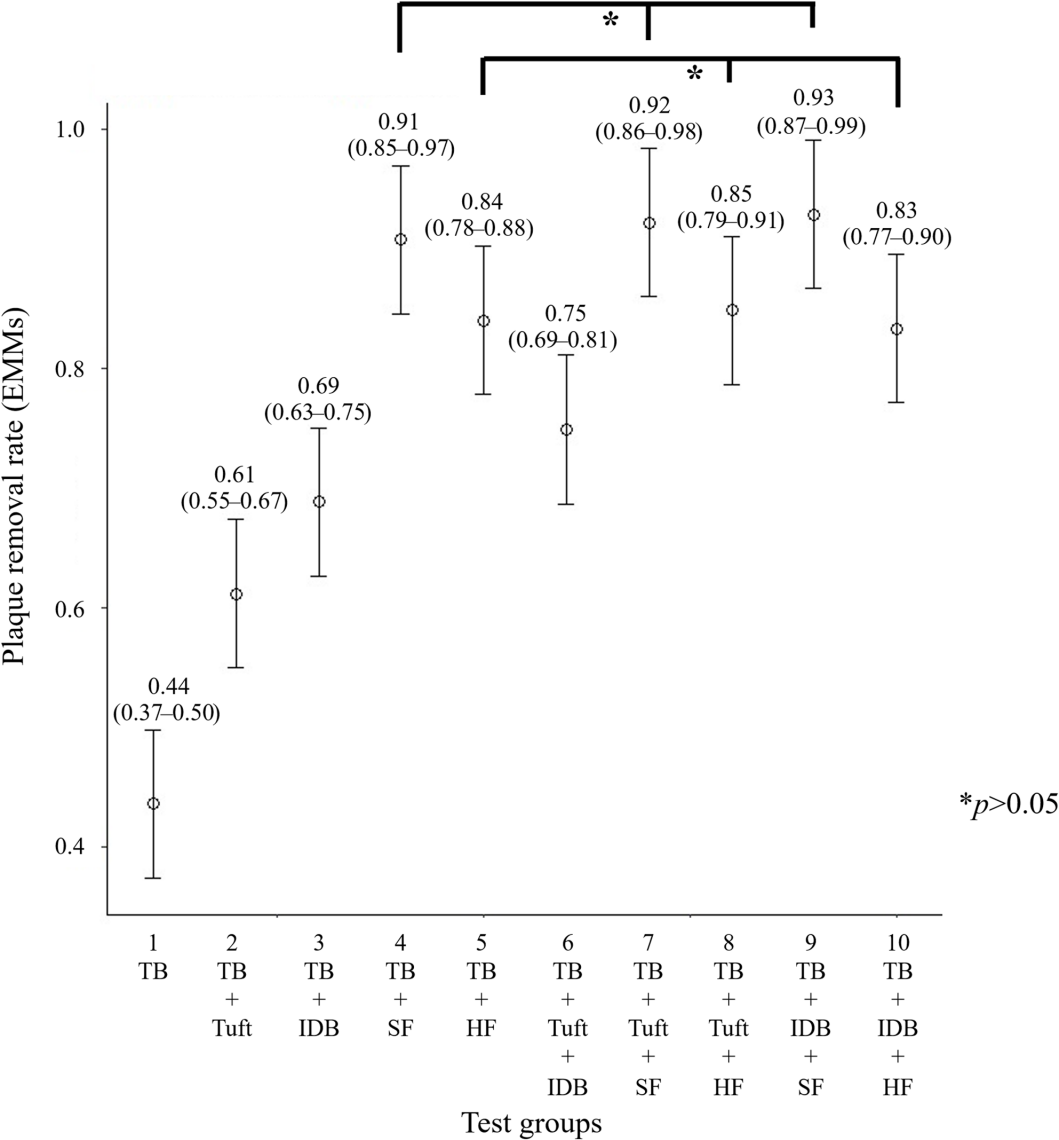
Estimated marginal means (EMMs) of artificial plaque removal rates. Numbers correspond to the 10 oral hygiene device combinations shown in Fig. 2. *No statistically significant differences among the string floss (SF)-containing groups or among the Y-shaped holder floss (HF)-containing groups (Tukey-adjusted *p* > 0.05)

The distribution of EMMs is shown in Fig. 3. Removal rates exceeding 90% were achieved in all combinations that included SF. Multiple comparison analysis indicated that the groups including SF exhibited significantly higher removal rates compared to the non-SF groups. The TB + Tuft + IDB protocol demonstrated intermediate plaque removal efficacy, exceeding that of the dual-combination groups without floss but remaining lower than that of the HF- and SF-containing protocols. Of all protocols tested, the combination of TB, IDB, and SF demonstrated the highest plaque removal rate. Under the conditions of our study, no statistically significant differences were observed among the SF-containing groups (TB + SF, TB + Tuft + SF, TB + IDB + SF) or among the HF-containing groups (TB + HF, TB + Tuft + HF, TB + IDB + HF; *p* > 0.05).

## Discussion

We systematically evaluated the mechanical plaque removal efficacy of manual toothbrushes used in combination with various oral hygiene devices under standardized experimental conditions. All combination groups demonstrated significantly higher removal rates compared to the TB group. In particular, protocols incorporating SF demonstrated removal rates exceeding 90%.

To conduct these evaluations, we adopted an in vitro design that enabled highly standardized experimental conditions in accordance with established protocols. Key features included strict control of tooth morphology, dentition, periodontal tissue configuration, and intraoral environmental variability, including the absence of saliva. The use of a specialized artificial plaque ensured uniform adhesion across all interdental surfaces. Furthermore, by distinguishing the gingival margin from the alveolar bone line, the evaluation area was standardized, addressing ambiguities in area definition reported in previous studies [20–22]. These design features enabled assessment of the mechanical accessibility and adaptability of each instrument to the tooth surface under controlled conditions.

Consequently, our study provides foundational data on the theoretical mechanical plaque removal performance achievable under standardized in vitro conditions, rather than direct evidence of clinical effectiveness.

To enhance the robustness of the findings, five trained dental professionals were included rather than a single operator or a mechanical simulator. In addition, a linear mixed-effects model was applied to account statistically for operator-specific variability. By incorporating inter-operator differences as a random effect, comparisons among cleaning protocols were estimated while accounting for the hierarchical structure of repeated measurements within operators. Accordingly, the observed removal rates were interpreted primarily as attributable to the cleaning protocols rather than to differences in operator performance. Although complete randomization of procedural order was not implemented, operators were assigned to different starting sequences, and inter-operator variability was adjusted for within the mixed-effects model. Nevertheless, a potential learning effect cannot be entirely excluded.

The baseline removal rate for TB was 0.44, aligning with previous reports that interproximal efficacy for manual brushes typically remains below 50% [5]. In contrast, every group incorporating adjunctive oral care tools demonstrated significantly higher removal rates (*p* < 0.001). To translate these findings into clinical outcomes, patient-related factors must be considered. Thomassen et al. [14] reported that the effectiveness of interdental cleaning at home depends on device performance, patient motivation, manual dexterity, and individual anatomical characteristics. Although SF demonstrated superior access in the experimental model, flossing is technically challenging for many users in the general population, and sustained adherence and correct use are frequently suboptimal. Therefore, selection of interdental cleaning devices in clinical practice should not be based on theoretical cleaning efficacy. Instead, clinicians should consider individual patient abilities and preferences and recommend approaches that can be maintained consistently over the long term.

Notably, all combinations involving dental floss achieved removal rates exceeding 90%. While this differs from some systematic reviews suggesting that IDBs are generally more effective for plaque control [8], it supports research indicating that floss is superior in specific anatomical contexts, such as the narrow interdental spaces where IDB insertion is restricted [9]. This is consistent with findings by Nakao et al., who reported that dental floss is highly effective for individuals with healthy periodontal tissues, whereas IDBs are more advantageous in the presence of deep periodontal pockets [9].

However, the interdental morphology varies considerably across different sites in the oral cavity. Root surface concavities, interdental space morphology, and contact tightness differ between anterior and posterior regions, as well as between mesial and distal surfaces. In our study, SF demonstrated high efficacy at tight contact points in the molar restoration model; however, caution is needed when extrapolating these findings to regions with wider interdental spaces or more complex root surface morphology.

The exceptionally high plaque removal rates observed in our study were notable even in comparison with previous in vitro studies using comparable standardized models. Previous studies have indicated that interdental plaque removal rates are generally approximately 20–60% [16–19]. In contrast, our study demonstrated removal rates of 60% or higher across all combination groups. Notably, all SF-containing groups achieved removal rates exceeding 90%, with EMMs of 0.91 (95% CI: 0.85–0.97) for TB + SF, 0.92 (95% CI: 0.86–0.98) for TB + Tuft + SF, and 0.93 (95% CI: 0.87–0.99) for TB + IDB + SF. In contrast, none of the HF-containing groups exceeded 90%, with EMMs of 0.83–0.85. These findings may reflect the high sensitivity of the present experimental model for detecting differences in mechanical accessibility of instruments under the constrained anatomical conditions of molar mesial surfaces. The observed differences may be attributed to the limited adaptability of holder-type floss, in which fixed tension reduces conformity to tooth surface morphology and subgingival contour, compromising access. Consistent with this interpretation, image analysis demonstrated residual plaque accumulation near the buccal and lingual gingival margins in the holder-type floss group.

Although the triple combination of TB + IDB + SF exhibited the highest nominal EMM (0.93), the difference was not statistically significant compared to the other SF-containing protocols (TB + SF and TB + Tuft + SF). This suggests that under the constrained anatomical conditions of the narrow interdental spaces in this model, the single addition of SF may have already reached a ceiling effect in mechanical plaque removal, leaving limited room for further cumulative efficacy by adding other devices. Clinical recommendations should therefore account for patient adherence, manual dexterity, and the long-term sustainability of interdental cleaning behaviors rather than focusing solely on these nominal numerical increases.

This study has several limitations. First, all cleaning procedures were performed by trained dental professionals under highly standardized conditions. Therefore, the present findings should be interpreted as reflecting the theoretical upper limit of mechanical plaque removal performance rather than the actual effectiveness achievable in routine self-care by the general population. Second, the study was conducted under controlled in vitro conditions that did not reproduce important intraoral factors such as saliva, soft tissue interference, inflammation, and biofilm maturation. In particular, the high plaque removal rates observed in this study should be interpreted cautiously. Previous studies have demonstrated that the presence of saliva can reduce cleaning efficiency, potentially because of its lubricating effect between the instrument and tooth surface [23]. Accordingly, the removal rates reported here may overestimate those achievable under clinical conditions. Third, the present experimental model was based on limited anatomical conditions and did not fully represent the diversity of interdental morphology encountered clinically, including variations in embrasure configuration, interdental spacing, and periodontal status. Therefore, caution is warranted when generalizing these findings to broader clinical populations. Finally, although the TB + IDB + SF protocol demonstrated the nominal highest mechanical cleaning performance in this experimental model, the simultaneous use of multiple adjunctive devices at a single site may not be practical or sustainable in routine home care. Thus, the present findings should be interpreted primarily as comparative baseline data regarding the mechanical accessibility of each cleaning protocol under controlled conditions, rather than as direct clinical recommendations.

Future in vivo studies and clinical trials incorporating patient-related factors, behavioral adherence, and long-term clinical outcomes are warranted.

## Conclusion

Our study demonstrated that, under standardized in vitro conditions, protocols incorporating interdental cleaning instruments significantly improved interdental plaque removal compared to TB. Furthermore, differences in removal efficacy were observed depending on the combination of instruments. Under the conditions of this experiment, the protocols that included SF demonstrated higher mechanical accessibility and greater plaque removal compared to other combinations. Future studies evaluating different anatomical conditions and general users are needed.

## Data Availability

The datasets used and/or analyzed during the current study are available from the corresponding author on reasonable request.

## Abbreviations

EMM: Estimated marginal mean
IDB: Interdental brush
TB: Toothbrush alone
Tuft: Single-tufted brush
SF: String floss
HF: Y-shaped holder dental floss
CI: Confidence Interval
